# Arsenic and Heavy Metal Accumulation and Risk Assessment in Soils around Mining Areas: The Urad Houqi Area in Arid Northwest China as an Example

**DOI:** 10.3390/ijerph15112410

**Published:** 2018-10-30

**Authors:** Shuai Song, Yuanjie Li, Lin Li, Maoyong Liu, Jing Li, Liang Wang, Chao Su

**Affiliations:** 1 State Key Laboratory of Urban and Regional Ecology, Research Center for Eco-Environmental Sciences, Chinese Academy of Sciences, Beijing 100085, China; shuaisong@rcees.ac.cn; 2 Inner Mongolia Institute of Geological Environmental Monitoring, Hohhot 010020, China; 18766953468@163.com; 3 Inner Mongolia Autonomous Region Metallurgy Research Institute, Hohhot 010010, China; lyjieouc@163.com (L.L.); frysoo@163.com (M.L.); rcees_ss@163.com (L.W.); 4 Key Laboratory of Ecosystem Network Observation and Modeling, Institute of Geographic Sciences and Natural Resources Research, Chinese Academy of Sciences, Beijing 100101, China; jingli@igsnrr.ac.cn; 5 Institute of Loess Plateau, Shanxi University, Taiyuan 030006, China

**Keywords:** mineral mining, metal contamination, risk assessment

## Abstract

Mining activities make important contributions to economic growth, but they can also produce massive amounts of solid waste, such as tailings and metal accumulations. Taking the Urad Houqi mining area in Inner Mongolia as the study area, this study systematically assessed the contamination risk of arsenic and heavy metals in the soils of the study area and explored the contamination characteristics in a key polymetallic mining area. For the whole study area, based on the Nemerow comprehensive pollution method, almost half of the investigated sites were contaminated, and the most contaminated site was Urad Houqi Qianzhen Mineral Concentration Co., Ltd. (Bayannaoer, China), a cooperation between the lead and zinc mining industry. The assessment results indicated that Cd and As were the elements of greatest concern, followed by Pb, Cr and Hg. Particularly, for the typical Dongshengmiao mining area, when compared with the GB15618-1995 standard values, As, Zn and Cd posed the most serious contamination threat, while Cr and Ni exhibited clean conditions. In addition, the vertical distribution maps demonstrated that the contents of arsenic and metals in some soil profiles were correlated with sampling depth. Therefore, arsenic and heavy metals pose high threat to soil ecosystems in this area, there is encouragement for some control and remediation measures to be taken into effect.

## 1. Introduction

The exploitation of mineral resources has great negative impacts not only on the surrounding soils but also on the total environment, and one of the most distinct impacts is metal contamination [1,2,3]. Anthropogenic activities, i.e., natural metal mineral mining and metal substance production, are the dominant sources of metal contamination in the environment, including soils [4,5,6,7], which could lead to a higher metal content in soils around the metallogenic belt, and the impacts of quartzite on soil metal contents are larger than those of carbonate rock [8].

During exploitation, large quantities of wastewater, waste gases and solid wastes are produced by mining activities, which are the main pathways of entry of metals into the surrounding soils [9,10,11]. A large number of metals are contained in the waste rock of tailing products, and they cannot be managed and recycled [12,13]. When the surface wastewater seeps into the ground, metals remain in the aeration zones and even reach the satiety zones, which could influence soil and groundwater ecosystems and induce plant growth inhibition [12,14,15]. Developed regions, such as North America and Europe, have placed high emphasis on metal pollution caused by the mining and smelting industries, and the mechanisms and remediation methods of metal pollution have been studied in depth [16,17]. The revegetation rate of damaged land in mining areas has reached 75% in developed regions and only 13.3% in China [18]. However, despite the severe situation in China, locals still overlook the metal contamination and remediation of surrounding soils in mining areas.

Therefore, the assessment of soil metal contamination in mining areas is essential and also the foundation of soil remediation. Many investigators have carried out a large number of studies on soil metal contamination. There are some commonly used methods for assessing soil metal pollution, such as the single factor index method, synthetic index method, geoaccumulation index method, and fuzzy mathematical method [4,19,20,21]. However, most of these methods have certain limitations. We must identify an appropriate method or conduct an assessment via multiple methods according to actual conditions. Usually, the background value of a local soil environment or the standard for soil environmental quality are used as references to ensure the reasonability of an assessment.

The study area of Urad Houqi is in the Inner Mongolia Autonomous region in arid northwest China. Metal contamination in soils is of concern in the Inner Mongolia Autonomous region because it is one of most important mineral and mining bases in China, and it is one of the 14 key prevention and control provinces in China. The Urad Houqi area belongs to the city of Bayan Nur, which is a key nonferrous metal mining region in the Inner Mongolia Autonomous area. The metal accumulation characteristics in soil of Bayan Nur are distinct. Zhang et al. demonstrated that metal contents in soil and underground water increased each year due to the exploitation of metal mining [22]. The results of another study showed that the concentrations of toxic metals (Co, Ni, Fe, Mn, Ba, Cu, Cr, Pb, and Zn) in the topsoil of the Hetao Plain had significant positive correlations with each other, and their common source was the mining area in Langshan and the industrial production area in the city [22]. Moreover, areas where local lesions were concentrated usually overlapped areas with high levels of metals. The contents of Pb and As in the soil and underground water of the Hetao Plain were much higher than their respective background values owing to the exploitation of mineral resources [23].

Therefore, in this study, we took a mining area (Urad Houqi) as an example using multiple methods to evaluate the risk of arsenic and heavy metal contamination in soils. The objectives of this study are (1) to conduct an arsenic and heavy metal contamination assessment in soils and (2) to explore the characteristics of arsenic and heavy metal contamination in a key polymetallic mining area.

## 2. Materials and Methods

### 2.1. Case Study Area

The Urad Houqi area, which has a vast territory and various topographies, is located in northwest Inner Mongolia. There are various mining industries in the study area, including lead, copper, zinc, and sulfur mines. In the central part, there is a large amount of nonferrous metal mineral resources. In addition, a polymetallic metallogenic belt is located in the northern part of the study area, where nonferrous metal mineral resources exist in rich reserves (e.g., copper, zinc and iron sulfide mineral resources) and vary in type. Nonferrous metal mining is an important economic pillar industry in this region.

There are 14 key enterprises investigated in the study area, including 10 metal mining enterprises, two smelting enterprises and two chemical manufacturing enterprises. Among them, there are nine still in production, four enterprises that have stopped production, while the remaining one has moved to another location. More details on these enterprises can be found in Table S1. Most of the enterprises are near sensitive lands, which comprise agricultural land, grassland and residential areas.

### 2.2. Soil Sampling and Chemical Analysis

**Figure 1:**
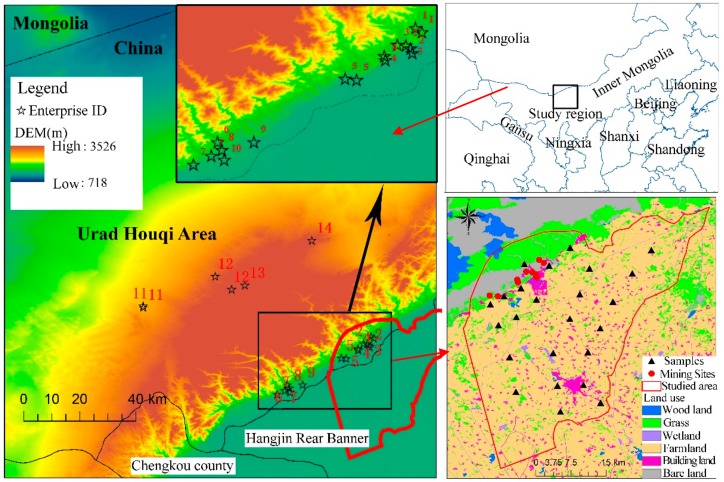
Location of different mining enterprises and sampling sites.

In this study, we set 14 sampling sites, consistent with the 14 investigated enterprises. Nine to twelve soil samples were collected from each site, including background and contaminated samples. Each sample was composed by five sub-samples that were collected from the center and four corners of an area of 50 × 50 m^2^. In total, 202 samples were collected from all sites, and 26 samples were collected to study the characteristics of Dongshengmiao mining area. Each background soil was collected from the earthwork stacking point formed before the enterprise was built. Each sample is analyzed separately. The location of the study area and investigated enterprises (sites) were shown in Figure 1. For the sites in plain areas, at least two samples were collected along the four cardinal directions of the producing tracts. For sites in mountainous areas, at least two samples were collected along three uniform angle directions of the producing tracts. The surface soil samples (0–20 cm) were collected from the sites. In addition, in order to study the vertical distribution and migration of arsenic and metals in soils of Dongshengmiao mining area, seven profile sampling sites were set in waste stacking location and tailing reservoir where arsenic and metals were easily to accumulate and transport downward to pose threat to the groundwater. Four-layer samples were collected from each profile sample (0–20 cm, 20–40 cm, 40–60 cm, and 60–100 cm layers). Approximately 1 kg of soil was collected from each layer.

The samples were preserved in sealed valve bags before they were dried by the vacuum freeze-drying method for 24 h. Then, they were crushed to pass through a 75 μm nylon mesh sieve. For the determination of arsenic and heavy metals, 0.5 g soil samples were weighed, placed into PVC digestion vessels, and then digested using 10 mL mixed-acid of HNO_3_ (Guaranteed Reagent, Tianjin Fenchuan Chemical Regent Technologies Co., Ltd., Tianjin, China), HClO_4_ (Guaranteed Reagent, Tianjin Zhengcheng Chemical Products Co., Ltd., Tianjin, China), HCl (Guaranteed Reagent, Tianjin Fenchuan Chemical Regent Technologies Co., Ltd., Tianjin, China) and HF (Guaranteed Reagent, Tianjin Fenchuan Chemical Regent Technologies Co., Ltd., Tianjin, China). Each analytical sample weight was between 0.10~2.00 g according to the content of target element. The digestion solution was diluted with 2% HNO_3_ to a final volume of 50 mL. The concentrations of Cr, Zn, Pb, Cu, Ni, and Cd in the soil samples were determined by inductively coupled plasma mass spectrometry (ICP-MS, Agilent 8800, Agilent Technologies Inc, Foster City, CA, USA). The concentrations of As and Hg were determined by atomic fluorescence spectrometer (AFS-2100, Beijing HaiGuang Instrument Ltd, Beijing, China). The quality control was assured by the analysis of duplicate samples and certified reference materials (GSS 13 and 17, purchased from the General Research Institute for Nonferrous Metals). According to the measurement of repeated samples and reference materials, the relative standard deviation (RSD) was below 3.6% for Cr, Zn, Pb, Cu, Ni, and Cd, and 7.3% for As and Hg, respectively. The recovery of reference materials was 84.4–125.7%.

### 2.3. Soil Arsenic and Heavy Metal Contamination Evaluation Standards and Methods

#### 2.3.1. Evaluation Factors

According to the soil environmental quality standard of China (GB15618-1995) and the technical specification for soil environmental monitoring (HJ166-2004), when combining the results of the experimental analysis, we selected mercury (Hg), arsenic (As), lead (Pb), chromium (Cr) and cadmium (Cd) as the evaluation factors. The reason is that they have been designated by the local government as key pollutants for prevention and control, as they are mainly derived from the non-ferrous metal mining and smelting industries in this region. Also, these substances of interest were selected because of their implications for human health based on toxicological and epidemiological data [24,25].

#### 2.3.2. Evaluation Methods

In this paper, we referred to the soil environmental quality standard of China (GB15618-1995) and the technical specification for soil environmental monitoring (HJ166-2004) to conduct the soil environmental quality assessment.

(1) Exceeding the standard rate

Exceeding the standard rate of element *i* (*R_i_*) was defined by the ratio of the number of samples exceeding the secondary guideline value in the soil environmental quality standard of China (GB15618-1995) (*S_i_*) to the total sample size (*S*) (Equation (1)). As a statistical indicator, the exceeding rates of the elements can be used to identify the main elements of contamination. The larger *R_i_* is, the more serious the contamination of element *i*:

(1) $$R_{i}=\frac{S_{i}}{S}\;\times \;100\%$$

where *R_i_* represents the exceeding standard rate of element *i*, *S_i_* represents the sample size of element *i* exceeding the secondary guideline value, and *S* represents the total sample size of element *i*.

(2) Single factor pollution index method

The single factor pollution index method is a typical and proven contamination assessment method. It is an index that reflects the influence of a single contaminant on soil. Its calculation is, as shown in Equation (2) [26]:

(2) $$P_{i}=\frac{C_{i}}{G_{i}}\;$$

where *P_i_* represents the single factor pollution index of element *i*, *C_i_* represents the content of element *i* in soils, and *G_i_* represents the guideline value of element *i* in soils.

(3) Nemerow comprehensive pollution index method

Nemerow comprehensive pollution index method is one of the most commonly used comprehensive assessment methods in soil metal contamination assessment [27]. This method was developed based on single pollution index. It allows the assessment of the overall degree of pollution in soils and includes the contents of all analyzed elements. Therefore, it is a comprehensive index reflecting the influences of multiple contaminants on the soil environment, as shown in Equations (3) and (4) [28]:

(3) $$P_{N}=\;\sqrt{\frac{{\bar{P}}^{2}+P^{2}{}_{max}}{2}}\;$$

(4) $$\bar{P}=\;\frac{1}{n}\sum_{i=1}^{n}P_{i}\;$$

where *P_N_* represents the Nemerow comprehensive pollution index, *P_i_* represents the single factor pollution index of element *i*, *P_max_* represents the maximum value of the single factor pollution index, and $\bar{P}$ represents the average value of the single factor pollution index.

#### 2.3.3. Evaluation Standard and Statistics Analysis

In this study, the Grade II values of GB15618-1995 were compared with the measured concentrations of metals in soils to conduct the evaluation, which are listed in Table 1. The grading standard for soil pollution is listed in Table 2.

Statistics analysis was carried out using SPSS 20.0 (IBM SPSS, Chicago, IL, USA), including statistical description (skewness, kurtosis, and standard deviation) and Spearman correlation analysis. The former was employed for examining the statistical distribution and central tendency of the data. Spearman correlation analysis was applied to calculate correlations between the heavy metal contents.

## 3. Results and Discussion

### 3.1. Soil Arsenic and Heavy Metal Pollution in Different Mining Enterprises

#### 3.1.1. Exceeding the Standard Rate

The statistical descriptions of Cr, As, Pb, Cd and Hg in soils for each site and their exceeding standard rates are listed in Table 3. Among all 13 sites (sites 2 and 3 are integrated into one investigated site because of the short distance), there were five sites where the contents of all five elements did not exceed the standard values (Table 3). However, in the other eight sites, Cd and As were identified as the main contaminants because the frequencies of their exceeding standard rates were higher, while the exceeding standard rates of Pb, Hg and Cr were almost zero (Table 3).

**Figure 2:**
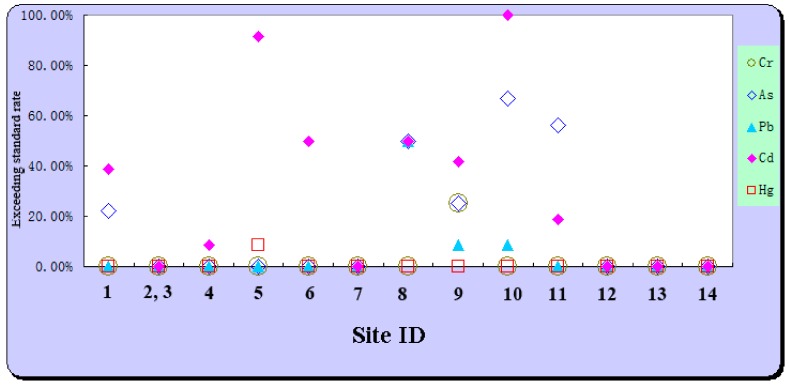
Exceeding the standard rate of arsenic and heavy metals in soils.

In addition, we made a scatter plot of the metal exceeding standard rates for each site. For most investigated sites, Cd and As were the main contaminants, followed by Pb (Figure 2). Generally, the metals of greatest concern, in order, would be as follows: Cd > As > Pb > Cr > Hg. However, there were some differences among the soil sites. Taking Hg as an example, only the exceeding standard rate of soil around Bayan Nur Zijin Nonferrous Metal Co., Ltd., Bayannaoer, China, (Site No.5), was greater than zero (8.33%). For Cr, only the exceeding standard rate of soil around Bayan Nur Feishang Copper Co., Ltd., Bayannaoer, China, (Site No.9), was greater than zero (25%). Notably, the exceeding standard rate of Cd in soil around Urad Houqi Yifengxi Chemistry Co., Ltd., Bayannaoer, China, (Site No.10) was 100%, which indicated that there might be potential risk of Cd contamination in the surrounding soil.

#### 3.1.2. Pollution Index Assessment

We also assessed the soil arsenic and heavy metal contamination using the single factor pollution index and Nemerow comprehensive pollution index methods, and the results are shown in Table 4. According to the single factor pollution index assessment results, the single factor pollution index values of Cd, As and Pb in soils in some sites were greater than 1.0, which exceeded the pollution index thresholds. These results indicated that the soils in some sites (i.e., Zhenyuan Mineral Concentration Factory, Bayan Nur Zijin Nonferrous Metal Co., Ltd., Inner Mongolia Qihua Mineral Concentration Factory, Urad Houqi Qianzhen Mineral Concentration Co., Ltd., Urad Houqi Yifengxi Chemistry Co., Ltd.) were seriously contaminated by Cd, As and Pb. The greater the single factor pollution index values were, the more serious the accumulation of metals.

For Cd, among the 13 investigated sites, there were five sites where the average single factor index values of the surrounding soils were greater than 1.0, which showed high accumulation of Cd in soil around these enterprises. The single factor index value for Urad Houqi Qianzhen Mineral Concentration Co., Ltd. (Site No.8 in Table 4), was the highest, with a value of 8.465. For As, there were 4 sites where the average single factor index value of the surrounding soils was greater than 1.0, among which the value at Urad Houqi Oubulage Copper Mineral Co., Ltd. (Site No.11), was extremely higher than those at other sites, with a value of 7.625. For Pb, there was only one site where the average single factor index value for the surrounding soil was greater than 1.0, with a value of 1.216, which indicated that the accumulation of Pb was generally minor.

Particularly, at some sampling sites, the single factor index values of As and Cd were relatively higher than those at other sites. The highest value of As was found in soil around Urad Houqi Oubulage Copper Mineral Co., Ltd. (Site No.11 in Table 4), with a value of 38.295, which substantially exceeded the pollution index threshold. The highest value of Cd was detected in the soil around Bayan Nur Zijin Nonferrous Metal Co., Ltd. (Site No.5 in Table 4), which reached up to 48.425, indicating high accumulation of Cd in the surrounding soil.

**Figure 3:**
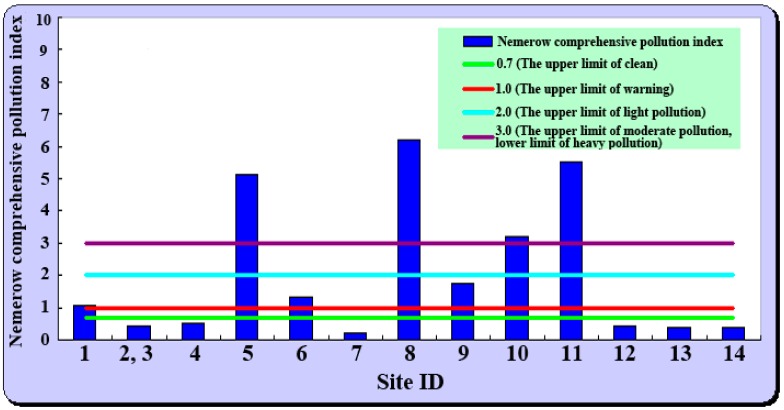
Soil arsenic and heavy metal pollution assessment by Nemerow comprehensive pollution method.

According to the Nemerow comprehensive pollution method, among the 13 investigated sites, there were four sites that exhibited levels of heavy pollution, three sites that exhibited levels of light pollution, and six sites that were clean (Figure 3). In other words, sites below the limit of warning accounted for 46.15% of the total sites, and 53.85% of the total sites were contaminated to varying degree ranges. Among the four heavily contaminated sites, the most contaminated site was at Urad Houqi Qianzhen Mineral Concentration Co., Ltd. (6.215), followed by Urad Houqi Oubulage Copper Mineral Co., Ltd. (5.523), with Cd and As being the dominant elements, respectively.

In a word, almost half of the investigated soils were contaminated by metals. Cd and As were the elements with the greatest concern, followed by Pb, Cr and Hg, even though there were some differences among the sites. These metals pose increasingly ecological and human health risk due to the bioaccumulation and biomagnification in the food chain [24,29]. The bioaccessibility of metals (i.e., Pb, As) in soils have been reported to be in the range of 0.1–68% [30]. The differences in the morphology, composition and mineralogy of metals may be the main reasons for the wide range of these values. In this study, the average value of Cd (1.03 mg/kg) exceeded the ecological screening levels in soils of 1.0 mg/kg for avian wildlife and 0.38 mg/kg for mammalian wildlife [31]. Food intake was the main route Cd entering the body, and the major threat to human health was chronic accumulation which could lead to kidney dysfunction, human carcinogen, and reproductive toxicity [32,33]. Since Cd is very biopersistent and its half-life period might reach as long as 30 years in the human body [34], the degree of contamination around mining enterprises indicated that atmospheric deposition and consequent accumulation in soils needed to be minimized. Besides, As values in nearly 15% of the total samples exceeded Grade II values of the Chinese standard, while the geometric average value of As was 3.84 times higher than the US baseline [24,35]. Arsenic compounds adsorb strongly to soils and could be transported over short distances to groundwater and crops (i.e., wheat and maize) [36]. Some studies showed that the contribution by aerosol inhalation was less important than by dust ingestion, while the daily oral intake of As surpassed the limitation for children in a mining region. Nevertheless, because long-term exposure to As was associated with skin damage, cancer risk, and urinary bladder [24], greater concern on this element was very important. Pb could accumulate in the entire food chain, and the risk of Pb poisoning through the food chain increased with the soil Pb level. Higher Pb concentrations were more likely to be found in leafy vegetables and root crops [25]. Though the average value of Pb (75.82 mg/kg) fell below the soil cleanup standard of 400 mg/kg for residential areas established by EPA [37], they were greater than the ecological soil screening levels of EPA for avian wildlife (16 mg/kg) and mammalian wildlife (59 mg/kg) [38]. Given the relatively widespread elevation in Pb levels, high Pb levels related to mining and smelting activities in the region might contribute to the exposure of local residents, especially for children. Moreover, Pb could cause serious injury to the brain, nervous system, and kidneys during the key periods of child growth [24,36]. Therefore, the metal pollution should be elevated as an important public health priority in the Urad Houqi region.

### 3.2. Source Apportionment

Nonferrous metal mining and smelting were the major sources of Cd and As contamination, which was similar with the study result of Li et al. [39]. Fundamentally, Cd and As are often associated with zinc, lead-zinc, and copper-lead-zinc deposits. In mining, smelting and roasting ores, Cd and As could be discharged into the surrounding environment through solid wastes (tailings, slag) [22,23], which led to the accumulation of Cd and As in the surrounding soil.

In particular, cadmium and zinc, lead often coexist in the nature. In this study area, it was associated with light-colored sphalerite with a larger reserve, which was similar with the research of Alloway [40]. During mining processes, after Pb and Zn are refined, more Cd leaves residue in tailings and broken ores, which can then be carried to additional areas due to artificial or natural causes, such as rainfall. For the smelting industry, before Cd is extracted from ores completely, Cd deposits into the surrounding soil along with particles in air during smelting activities [41], which makes the Cd concentration in the humus layer exceed the standard value. Finally, based on the accumulation assessment results, the mineral concentration industry plays a predominant role in soil pollution, followed by the smelting industry and acid manufacturing industry.

### 3.3. Soil Arsenic and Heavy Metal Pollution Characteristics in a Key Mining Area

#### 3.3.1. Statistical Characteristics of Arsenic and Heavy Metal Pollution in Surface Soils

In this section, we selected a typical area, the Dongshengmiao mining area, as a key case to explore soil arsenic and heavy metal pollution characteristics. The Dongshengmiao mining area includes three investigated enterprises (Urad Houqi Zijin Mining Co., Ltd., Inner Mongolia Dongshengmiao Mining Co., Ltd. and Wancheng Business Dongshengmiao Co., Ltd.), which shared the same tailings. This area was representative in exploring the characteristics of soil heavy metal pollution in polymetallic mining areas.

The statistical characteristics of Cr, Ni, Cu, Zn, As, Cd, and Pb are listed in Table 5. As, Zn and Cd emerged as posing the most serious contamination threat, while Cr and Ni were clean when compared with the GB15618-1995 standard values. The exceeding standard rates of Cr, Ni, Cu, Zn, As, Cd, and Pb were 0.00%, 0.00%, 7.69%, 30.77%, 50.00%, 26.92%, and 11.54%, respectively. In addition, Cd, Pb, As, Zn and Cu had larger coefficients of variation (CVs; >1.0), indicating that they were obviously affected by external interference and that the spatial distributions of these metals varied remarkably [42].

Moreover, there were significant positive correlations between metals Ni, Cu, Zn, As, Cd, and Pb, such as Cr and Ni, Ni and Cu, Zn and Cd, and Cd and Pb (*p* < 0.01) (Table 6), which showed that there may have been isogenesis or they were less affected by the soil parent materials [42]. Therefore, the mining activities in this key area made a large contribution to the accumulation of arsenic and heavy metals in the surrounding agricultural soils and the sedimentation of metals in the atmosphere.

#### 3.3.2. Spatial Distribution of Arsenic and Heavy Metal Accumulations

**Figure 4:**
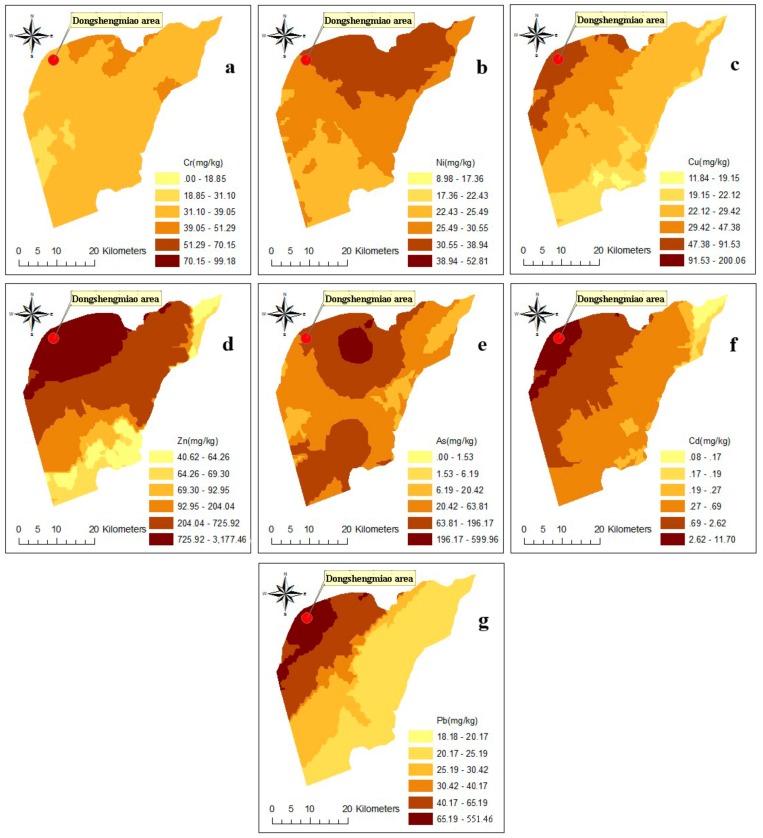
Spatial distribution of arsenic and heavy metal accumulations in soil in the Dongshengmiao mining area: (**a**) Cr, (**b**) Ni, (**c**) Cu, (**d**) Zn, (**e**) As, (**f**) Cd, and (**g**) Pb.

As one of the main geostatistical methods, kriging interpolation was used to draw the spatial distribution map of arsenic and heavy metal accumulations in the key mining area. This method demonstrated that the accumulations of Cu, Zn, Cd and Pb were the heaviest in the Dongshengmiao mining area and gradually became lighter from the mining area with distance (Figure 4). 

**Figure 5:**
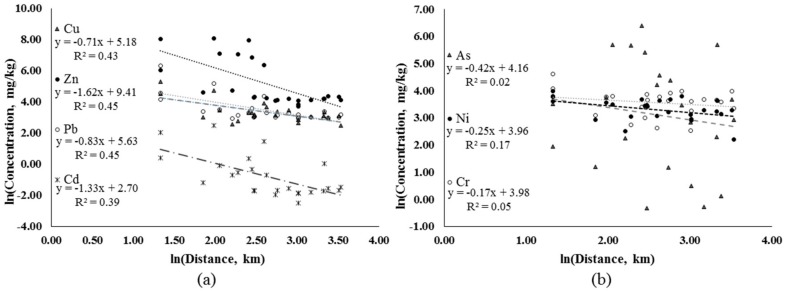
The relationship between element concentrations and distance to the mine: (**a**) Cu, Zn, Pb, and Cd, (**b**) As, Ni, and Cr.

The contents of Zn, Cd and As in the surrounding mining area exceeded the secondary standard values. The As content was higher downstream of the mining area and in northern Bayan Nur; this result was closely related to the high background value of sediments downstream of the mining area, which was the key area of As prevention and control. The higher As content in northern Bayan Nur was due to atmospheric deposition. However, Cr and Ni did not demonstrate apparent spatial distribution differences. The results showed significant logarithmic correlations between Cu, Zn, Pb and Cd concentrations and distance to the mines (Figure 5a, *p* < 0.05). However, no significant correlations were found between Cr, As and Hg concentrations and the distance (Figure 5b, *p* > 0.05). All the element concentrations showed a decrease trend with distance to the mine (Figure 5), which indicated that Cu, Zn, Pb and Cd in soils mainly originated from mining and smelting activities through short-distance transmission processes.

#### 3.3.3. Vertical Distribution of Soil Arsenic and Heavy Metal Accumulations

Seven soil profiles were set for the Dongshengmiao mining area, including the surrounding soils of a wastewater drainage ditch of a smelting plant, the front belt of a mining area, alluvial plains, and the surrounding agricultural soils of mining areas. Finally, four samples were taken from four layers (1: 0–20 cm; 2: 20–40 cm; 3: 40–60 cm; 4: 60–100 cm) for each profile (except profile B). The contents of the heavy metals in the samples are listed in Table 7. 

**Figure 6:**
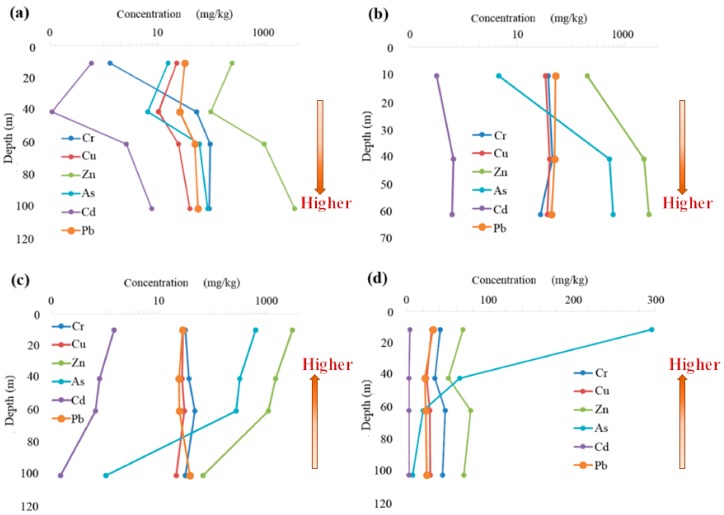
Vertical distributions of arsenic and heavy metal accumulations in typical profiles of the Dongshengmiao mining area: (**a**) profile A, (**b**) profile B, (**c**) profile C, (**d**) profile D.

We selected four typical profiles, A (Wastewater drainage ditch of the Zijin smelting plant), B (Front belt of a mining area), C (Surrounding agricultural soil of a mining area), and D (Surrounding agricultural soil of an alluvial plain), to make the vertical distribution maps of the arsenic and heavy metal contents (Figure 6).

Two vertical distribution features were explained by the arsenic and heavy metal contents in the surrounding soils of the key mining area. One feature was that the arsenic and heavy metal contents increased with sampling depth, which was characterized by the surrounding soils of the wastewater drainage ditch and front belt (Table 7 and Figure 6a,b), where the transport of arsenic and heavy metals was driven by water. In this case, the element contents below the surface layer were higher. The contents of Zn, Cd and As exceeded the tertiary values of GB15618-1995. The metals were transported downward with the water flow under the influences of wastewater and irrigation and then accumulated at the bottom. In particular, the contents of Zn in soil layer 4 (60–100 cm) of the wastewater drainage ditch and front belt were approximately 10-fold higher than those in the surface layer (Table 7), indicating that the underground water was potentially threatened.

On the other hand, the element contents decreased with the sampling depth (Figure 6c,d). In this case, the profiles were far from the surface water and mines. The elements (Cd, As, Zn) accumulated predominantly in the surface indicating that the soils were seriously contaminated by exogenous pollution sources like atmospheric deposition and industrial, agricultural and domestic activities. And they transported downward very slowly owing to far from the surface water. In the alluvial plains far from the mines, the element contents in soils were obviously lower than those in the surrounding soils of the mines, and their vertical variations were relatively smaller. However, they were affected in several ways, such as by element contents in the surface layer, land use types, industrial and agricultural activities, atmospheric deposition and wind direction. In addition, for each metal, we analyzed its accumulation characteristics in the four typical profiles. Generally, there were no large differences in the average contents of Cr, Zn, As, Cd and Pb in the four profiles (*p* > 0.05) except for Ni and Cu. For Ni, the average contents of profiles C and D were significantly higher than profile B, which was significantly higher than that of profile A (*p* < 0.05). For Cu, the average contents of profiles B, C and D were significantly higher than that of profile A (*p* < 0.05), while there were no significant differences among profiles B, C and D (*p* > 0.05). The contents of Cr and Cu were generally higher in the middle layers than those in the surface and bottom layers. It demonstrated that Cr and Cu showed surface-aggregation property to a certain level and they might transport downward very slowly because of the arid climate and less rainfall in Inner Mongolia [43]. However, all the contents of Cr, Cu and Ni in the four profiles did not exceed the standard values, posing less risk to soil ecosystems. The maximum values of both Zn and As were found in the surface layer of the surrounding agricultural soil of the mining area (profile C), and they both exceeded the tertiary values of GB15618-1995, which showed that the surrounding agricultural soil of the mining area was seriously contaminated by Zn and As. The highest content of Cd was detected in the bottom layer of profile A, followed by the surface and middle layers of profile C. Because the three enterprises included in this key area belonged to lead and zinc smelting/mining industry (Table S1). And, cadmium and zinc, lead often coexist in the nature [44]. Some scientists also found that the unreasonable exploitation of lead and zinc mines could bring about the contamination of Cd in the North America, North Europe and East Asia [45,46,47]. The highest contents of Cd exceeded the second value of GB15618-1995 in this area, indicating that it suffered from intense Cd pollution. However, the surrounding agricultural soil of the alluvial plain (profile D) was not contaminated by heavy metals.

## 4. Conclusions

Mining activities not only lead to the massive stacking of tailings, which was one of the six most concerned solid wastes listed in “The 13th five-year plan for comprehensive utilization of industrial solid wastes” in China, but also cause heavy metal contamination. Inner Mongolia is one of the mineral resource bases in China. This study used the Urad Houqi mining area as the study area, conducted a soil arsenic and heavy metal contamination assessment for the entire area, and explored the characteristics of soil arsenic and heavy metal contamination in the Dongshengmiao mining area, which is a typical polymetallic mining area. In general, almost half of the investigated sites were contaminated by Cd, As and Pb, with the mineral concentration industry, smelting industry and acid manufacturing industry being the dominant sources. Particularly, for the Dongshengmiao mining area, As, Zn and Cd posed the most serious contamination risks, followed by Pb, and the accumulations of these metals was the heaviest in the mining area, which gradually decreased with distance. Therefore, increased concerns and control measures are needed for As, Cd, Pb and Zn contamination in the Urad Houqi mining area.

## Figures and Tables

**Table 1:** Grade II values of GB15618-1995 (unit: mg/kg).

Element pH	Cr		As		Pb	Cd	Hg
	CEC > 5 cmol(+)/kg	CEC ≤ 5 cmol(+)/kg	CEC > 5 cmol(+)/kg	CEC ≤ 5 cmol(+)/kg			
<6.5	150	75	40	20	250	0.3	0.3
6.5–7.5	200	100	30	15	300	0.3	0.5
>7.5	250	125	25	12.5	350	0.6	1

Note: CEC represents cationic exchange capacity.

**Table 2:** Grading standard for soil pollution of arsenic and metals.

Grade	Single Factor Pollution Index	Nemerow Comprehensive Pollution Index	Level
1	*P_i_* ≤ 0.7	*P_N_* ≤ 0.7	Clean
2	0.7 < *P_i_* ≤ 1.0	0.7 < *P_N_* ≤ 1.0	Warning
3	1.0 < *P_i_* ≤ 2.0	1.0 < *P_N_* ≤ 2.0	Light pollution
4	2.0 < *P_i_* ≤ 3.0	2.0 < *P_N_* ≤ 3.0	Moderate pollution
5	*P_i_* > 3.0	*P_N_* > 3.0	Heavy pollution

**Table 3:** Description of Cr, As, Pb, Cd and Hg in soils (0–20 cm) for each site and their exceeding standard rates (dry weight).

Site ID (Enterprise Name, Number of Samples)		Soil Arsenic and Heavy Metal Content (mg/kg)				
		Cr	As	Pb	Cd	Hg
	Recommended Value	250.000	25.000	350.000	0.600	1.000
1 (Zhenyuan Mineral Concentration Factory, Bayannaoer, China, 18)	Average	49.100	18.936	84.167	0.454	0.014
	Maximum	67.400	131.740	176.600	1.127	0.022
	Minimum	24.600	4.700	23.900	0.133	0.009
	Median	50.750	13.150	85.000	0.402	0.014
	SD **	13.400	28.478	50.739	0.296	0.004
	Exceeding rate	0.00%	22.22%	0.00%	38.89%	0.00%
2, 3 * (Urad Houqi Zijin Mining Co., Ltd., Wancheng Business Dongshengmiao Co., Ltd., Bayannaoer, China, 30)	Average	87.845	11.263	32.885	0.232	0.019
	Maximum	105.800	14.910	43.300	0.339	0.031
	Minimum	58.100	7.150	25.900	0.161	0.012
	Median	88.250	11.100	31.700	0.218	0.018
	SD **	15.151	2.456	4.023	0.054	0.006
	Exceeding rate	0.00%	0.00%	0.00%	0.00%	0.00%
4 (Inner Mongolia Dongshengmiao Mining Co., Ltd., Bayannaoer, China, 12)	Average	60.933	11.498	35.842	0.383	0.030
	Maximum	74.000	14.620	56.100	0.720	0.048
	Minimum	51.500	7.400	25.800	0.208	0.020
	Median	59.500	12.550	31.400	0.374	0.028
	SD **	8.449	2.445	10.249	0.137	0.008
	Exceeding rate	0.00%	0.00%	0.00%	8.33%	0.00%
5 (Bayan Nur Zijin Nonferrous Metal Co., Ltd., Bayannaoer, China, 12)	Average	49.108	10.359	48.792	4.246	0.327
	Maximum	66.500	17.050	137.500	29.055	2.253
	Minimum	40.000	8.840	25.700	0.460	0.014
	Median	49.450	9.655	36.000	1.326	0.047
	SD **	7.218	2.227	31.960	7.936	0.660
	Exceeding rate	0.00%	0.00%	0.00%	91.67%	8.33%
6 (Inner Mongolia Qihua Mineral Concentration Factory, Bayannaoer, China, 12)	Average	30.464	6.403	61.750	1.047	0.023
	Maximum	51.300	9.810	163.100	3.494	0.069
	Minimum	10.200	4.670	24.900	0.202	0.009
	Median	30.850	5.500	52.600	0.391	0.015
	SD **	16.482	1.793	38.359	1.052	0.018
	Exceeding rate	0.00%	0.00%	0.00%	50.00%	0.00%
7 (Inner Mongolia Qihua Sulfuric Acid Factory, Bayannaoer, China, 12)	Average	22.086	5.874	19.600	0.128	0.012
	Maximum	24.700	9.470	22.900	0.171	0.019
	Minimum	17.300	4.640	17.400	0.086	0.007
	Median	22.800	5.360	19.000	0.112	0.012
	SD **	2.490	1.624	1.776	0.032	0.004
	Exceeding rate	0.00%	0.00%	0.00%	0.00%	0.00%
8 (Urad Houqi Qianzhen Mineral Concentration Co., Ltd., Bayannaoer, China, 18)	Average	27.950	19.890	323.900	2.596	0.055
	Maximum	41.100	44.510	999.600	6.726	0.118
	Minimum	11.600	6.600	31.400	0.221	0.014
	Median	29.050	16.065	194.250	1.868	0.044
	SD **	10.541	14.417	367.486	2.700	0.042
	Exceeding rate	0.00%	50.00%	50.00%	50.00%	0.00%
9 (Bayan Nur Feishang Copper Co., Ltd., Bayannaoer, China, 12)	Average	71.083	12.042	120.783	1.366	0.056
	Maximum	248.100	33.910	508.600	4.839	0.195
	Minimum	17.200	4.820	28.100	0.159	0.014
	Median	23.600	7.735	53.750	0.440	0.029
	SD **	89.086	9.189	143.238	1.724	0.055
	Exceeding rate	25.00%	25.00%	8.33%	41.67%	0.00%
10 (Urad Houqi Yifengxi Chemistry Co., Ltd., Bayannaoer, China, 12)	Average	26.483	28.122	163.042	1.465	0.028
	Maximum	41.500	52.580	397.400	2.646	0.042
	Minimum	15.900	9.570	89.400	0.921	0.011
	Median	23.650	24.835	135.800	1.352	0.028
	SD **	8.140	15.826	77.725	0.518	0.011
	Exceeding rate	0.00%	66.67%	8.33%	100.00%	0.00%
11 (Urad Houqi Oubulage Copper Mineral Co., Ltd., Bayannaoer, China, 16)	Average	38.856	110.896	26.613	0.289	0.022
	Maximum	57.900	478.690	68.000	0.959	0.053
	Minimum	22.400	13.560	15.500	0.085	0.013
	Median	38.300	29.900	22.000	0.143	0.016
	SD **	8.496	155.524	13.288	0.276	0.012
	Exceeding rate	0.00%	56.25%	0.00%	18.75%	0.00%
12 (Bayan Nur West Copper Co., Ltd., Bayannaoer, China, 16)	Average	29.256	7.789	27.717	0.080	0.010
	Maximum	43.800	10.010	38.900	0.139	0.016
	Minimum	21.900	6.190	18.100	0.059	0.008
	Median	27.600	7.320	27.500	0.076	0.009
	SD **	6.429	1.122	5.702	0.019	0.002
	Exceeding rate	0.00%	0.00%	0.00%	0.00%	0.00%
13 (Urad Houqi Xinxing Mining Co., Ltd., Bayannaoer, China, 16)	Average	39.700	9.655	23.000	0.080	0.015
	Maximum	67.900	13.580	34.700	0.135	0.022
	Minimum	16.600	5.210	14.400	0.049	0.010
	Median	34.900	10.380	22.400	0.073	0.015
	SD **	18.522	2.648	5.806	0.027	0.004
	Exceeding rate	0.00%	0.00%	0.00%	0.00%	0.00%
14 (Urad Houqi Ebutu Nickel Mineral Co., Ltd., Bayannaoer, China, 16)	Average	38.115	7.281	17.608	0.067	0.012
	Maximum	54.100	8.460	18.700	0.084	0.015
	Minimum	32.300	6.170	16.100	0.045	0.009
	Median	35.200	7.240	17.600	0.065	0.011
	SD **	6.363	0.740	0.837	0.012	0.002
	Exceeding rate	0.00%	0.00%	0.00%	0.00%	0.00%

Note: * Sites 2 and 3 are too near and they are located in the same gully region, so they are integrated into one investigated site. ** SD means standard deviation.

**Table 4:** Soil arsenic and heavy metal pollution assessment results by pollution index methods.

Site ID		Single Factor Pollution Index Value					Nemerow Comprehensive Pollution Index	Result
		Cr	As	Pb	Cd	Hg		
1	Maximum	0.295	10.539	0.334	1.097	0.015	7.652	Heavy pollution
	Minimum	0.246	0.527	0.068	0.222	0.019	0.403	Clean
	Average	0.302	1.287	0.243	0.785	0.015	1.084	Light pollution
2, 3	Maximum	0.667	0.746	0.092	0.332	0.019	0.589	Clean
	Minimum	0.377	0.322	0.091	0.365	0.019	0.314	Clean
	Average	0.373	0.467	0.095	0.404	0.020	0.408	Clean
4	Maximum	0.206	0.443	0.160	1.200	0.048	0.897	Warning
	Minimum	0.248	0.496	0.077	0.385	0.028	0.392	Clean
	Average	0.244	0.460	0.102	0.638	0.030	0.517	Clean
5	Maximum	0.198	0.390	0.215	48.425	2.253	35.007	Heavy pollution
	Minimum	0.162	0.377	0.080	0.767	0.021	0.577	Clean
	Average	0.213	0.451	0.139	7.077	0.327	5.140	Heavy pollution
6	Maximum	0.386	0.785	0.466	5.823	0.039	4.252	Heavy pollution
	Minimum	0.138	0.406	0.071	0.337	0.009	0.317	Light pollution
	Average	0.211	0.440	0.178	1.790	0.024	1.330	Light pollution
7	Maximum	0.166	0.415	0.054	0.178	0.011	0.316	Clean
	Minimum	0.099	0.206	0.058	0.187	0.012	0.166	Clean
	Average	0.100	0.265	0.056	0.214	0.012	0.220	Clean
8	Maximum	0.531	2.226	3.998	22.420	0.394	16.396	Heavy pollution
	Minimum	0.091	0.384	0.090	0.462	0.024	0.359	Clean
	Average	0.289	1.152	1.216	8.465	0.139	6.215	Heavy pollution
9	Maximum	1.985	2.713	1.453	7.908	0.195	5.944	Heavy pollution
	Minimum	0.145	0.388	0.114	0.430	0.014	0.341	Clean
	Average	0.561	0.937	0.345	2.277	0.056	1.765	Light pollution
10	Maximum	0.444	1.604	1.185	8.820	0.140	6.471	Heavy pollution
	Minimum	0.210	1.279	0.311	1.535	0.028	1.185	Light pollution
	Average	0.261	1.613	0.564	4.315	0.066	3.202	Heavy pollution
11	Maximum	0.314	38.295	0.118	1.250	0.046	27.664	Heavy pollution
	Minimum	0.159	0.592	0.065	0.190	0.015	0.443	Light pollution
	Average	0.207	7.625	0.076	0.481	0.022	5.523	Heavy pollution
12	Maximum	0.218	0.738	0.111	0.232	0.010	0.554	Clean
	Minimum	0.161	0.380	0.059	0.128	0.013	0.288	Clean
	Average	0.207	0.558	0.079	0.133	0.010	0.419	Clean
13	Maximum	0.236	0.740	0.064	0.133	0.017	0.550	Clean
	Minimum	0.128	0.383	0.074	0.125	0.015	0.290	Clean
	Average	0.190	0.482	0.066	0.133	0.015	0.363	Clean
14	Maximum	0.282	0.677	0.049	0.107	0.015	0.505	Clean
	Minimum	0.138	0.286	0.047	0.107	0.011	0.219	Clean
	Average	0.259	0.488	0.050	0.112	0.012	0.369	Clean

**Table 5:** Statistical description of arsenic and heavy metals in surface soil in the Dongshengmiao mining area (dry weight).

Elements	Minimum (mg/kg)	Maximum (mg/kg)	Average (mg/kg)	SD	CV	Skewness	Kurtosis
Cr	-	99.18	37.21	19.10	0.51	1.00	3.54
Ni	8.98	52.81	29.25	10.52	0.36	0.17	−0.29
Cu	11.84	200.06	37.18	40.23	1.08	3.18	11.05
Zn	40.62	3177.46	564.88	960.39	1.70	2.07	3.14
As	-	599.96	84.24	142.83	1.70	2.38	6.07
Cd	0.08	11.70	1.30	2.65	2.04	3.18	10.32
Pb	18.18	551.46	56.13	106.64	1.90	4.38	20.38

Note: SD and CV indicate standard deviation and coefficient of variation, respectively.

**Table 6:** Spearman correlation analysis between elements.

Elements	Cr	Ni	Cu	Zn	As	Cd	Pb
Cr	1.000	0.748 **	0.594 **	0.077	0.028	0.084	0.229
Ni	-	1.000	0.805 **	0.341 *	0.399 *	0.423 *	0.534 **
Cu	-	-	1.000	0.542 **	0.447 *	0.644 **	0.785 **
Zn	-	-	-	1.000	0.503 **	0.858 **	0.599 **
As	-	-	-	-	1.000	0.633 **	0.545 **
Cd	-	-	-	-	-	1.000	0.795 **
Pb	-	-	-	-	-	-	1.000

Note: ** Correlation is significant at the 0.01 level. * Correlation is significant at the 0.05 level.

**Table 7:** The contents of arsenic and heavy metals in the samples for each profile (dry weight, unit: mg/kg).

Location/Profile	ID	Cr	Ni	Cu	Zn	As	Cd	Pb
Wastewater drainage ditch of the Zijin smelting plant (A)	A-1	0.00	12.09	13.09	112.47	9.40	0.48	18.18
	A-2	28.44	8.20	6.51	49.02	4.26	0.11	14.97
	A-3	48.54	13.20	14.14	386.56	31.71	1.89	26.56
	A-4	46.34	19.83	22.01	1254.16	43.41	5.03	30.01
Front belt of a mining area (B)	B-1	22.07	18.39	19.99	100.69	3.26	0.30	29.19
	B-2	26.25	25.49	23.54	908.94	236.21	0.57	28.59
	B-3	16.50	21.95	21.39	1087.89	272.06	0.53	25.31
Surrounding agricultural soil of a mining area (C)	C-1	29.55	39.27	27.08	2837.19	599.96	1.44	26.60
	C-2	34.50	36.69	25.92	1393.44	300.86	0.77	22.67
	C-3	45.00	39.77	28.56	1032.84	261.86	0.65	23.10
	C-4	29.96	26.26	20.24	63.72	0.00	0.15	36.42
Surrounding agricultural soil of an alluvial plain (D)	D-1	37.80	37.79	27.48	65.16	293.21	1.02	29.30
	D-2	31.50	32.90	21.63	47.67	61.22	0.13	19.44
	D-3	43.74	32.76	25.40	74.86	16.89	0.15	20.40
	D-4	40.40	31.38	25.66	66.18	4.72	0.16	21.66
Surrounding agricultural soil of an alluvial plain (E)	E-1	53.70	44.39	31.88	66.99	31.79	0.21	25.79
	E-2	52.05	44.18	32.82	67.08	59.13	0.22	26.43
	E-3	37.65	48.32	36.03	74.45	47.36	0.21	28.58
	E-4	30.75	34.30	25.40	70.75	0.00	0.16	21.54
Surrounding forestry soil of an alluvial plain (F)	F-1	12.60	22.56	17.21	46.19	16.97	0.15	23.36
	F-2	17.10	49.52	19.02	40.22	64.29	0.12	23.45
	F-3	9.00	21.56	15.59	34.73	36.81	0.14	23.00
	F-4	5.85	27.20	22.31	65.72	8.54	0.25	23.12
Surrounding agricultural soil of a mining area (G)	G-1	15.30	21.02	15.69	1149.54	290.96	0.59	22.86
	G-2	16.20	26.01	20.13	1006.74	51.69	0.55	24.08
	G-3	43.50	35.69	27.74	1242.39	280.81	0.72	26.00
	G-4	33.15	30.42	29.18	1276.59	294.41	0.75	26.49

## Supplementary Materials

The following are available online at http://www.mdpi.com/1660-4601/15/11/2410/s1, Table S1: Summary of the 14 key investigated sites in Urad Houqi.
