# Supplementary material for: Arsenic and Heavy Metal Accumulation and Risk Assessment in Soils around Mining Areas: The Urad Houqi Area in Arid Northwest China as an Example
Source: Int J Environ Res Public Health. 2018 Oct 30;15(11):2410. doi: 10.3390/ijerph15112410 (PMC6267203; doi:10.3390/ijerph15112410)
Supplement: Supplementary file 1 [file ijerph-15-02410-s001.pdf]

Supplementary Materials

Arsenic and heavy metal accumulation and risk assessment in soils around mining areas: Taking the Urad Houqi area in arid northwest China as an example

Contents

Table S1 Summary of the 14 key investigated sites in Urad Houqi

Table S1 Summary of the 14 key investigated sites in Urad Houqi

Site ID	Enterprise Name	Location (Town)	Industry	Note
1	Zhenyuan Mineral Concentration Factory	Bayinbaolige Town	Lead and zinc mining	Production stopped
2	Urad Houqi Zijin Mining Co., Ltd	Bayinbaolige Town	Common nonferrous metal mining	
3	Wancheng Business Dongshengmiao Co., Ltd	Bayinbaolige Town	Lead and zinc mining	
4	Inner Mongolia Dongshengmiao Mining Co., Ltd	Bayinbaolige Town	Lead and zinc mining	
5	Bayan Nur Zijin Nonferrous Metal Co., Ltd	Bayinbaolige Town	Lead and zinc smelting	
6	Inner Mongolia Qihua Mineral Concentration Factory	Huhewenduer Town	Lead and zinc mining	
7	Inner Mongolia Qihua Sulfuric Acid Factory	Huhewenduer Town	Inorganic acid manufacturing	
8	Urad Houqi Qianzhen Mineral Concentration Co., Ltd	Huhewenduer Town	Lead and zinc mining	Production stopped
9	Bayan Nur Feishang Copper Co., Ltd	Huhewenduer Town	Copper smelting	
10	Urad Houqi Yifengxi Chemistry Co., Ltd	Huhewenduer Town	Inorganic acid manufacturing	Relocation
11	Urad Houqi Oubulage Copper Mineral Co., Ltd	Huogeqisumu	Copper mining	
12	Bayan Nur West Copper Co., Ltd	Huogeqisumu	Copper mining	
13	Urad Houqi Xinxing Mining Co., Ltd	Huogeqisumu	Copper mining	Production stopped
14	Urad Houqi Ebutu Nickel Mineral Co., Ltd	Chaogewenduer Town	Nickel and cobalt mining	Production stopped
